# Cell Fusion in the War on Cancer: A Perspective on the Inception of Malignancy

**DOI:** 10.3390/ijms17071118

**Published:** 2016-07-13

**Authors:** Jeffrey L. Platt, Xiaofeng Zhou, Adam R. Lefferts, Marilia Cascalho

**Affiliations:** Departments of Surgery and of Microbiology & Immunology, University of Michigan, A520B Medical Sciences Research Building I, 1150 W. Medical Center Drive, Ann Arbor, MI 48109-5656, USA; xiazhou@med.umich.edu (X.Z.); aleffert@umich.edu (A.R.L.); marilia@umich.edu (M.C.)

**Keywords:** cell fusion, cancer, malignant transformation, aneuploidy, DNA damage, oncogenesis, cancer progression, mitosis, mutation, immune surveillance

## Abstract

Cell fusion occurs in development and in physiology and rarely in those settings is it associated with malignancy. However, deliberate fusion of cells and possibly untoward fusion of cells not suitably poised can eventuate in aneuploidy, DNA damage and malignant transformation. How often cell fusion may initiate malignancy is unknown. However, cell fusion could explain the high frequency of cancers in tissues with low underlying rates of cell proliferation and mutation. On the other hand, cell fusion might also engage innate and adaptive immune surveillance, thus helping to eliminate or retard malignancies. Here we consider whether and how cell fusion might weigh on the overall burden of cancer in modern societies.

## 1. Introduction

In the “Annual message to Congress on the Sate of the Union” delivered on 22 January 1971, President Richard Nixon declared: “I will (ask for funds) to launch an intensive campaign to find a cure for cancer”, adding that “The time has come in America when the same kind of concentrated effort that split the atom and took man to the moon should be turned toward conquering this dread disease”. Whether this address actually declared a “war on cancer”, as some would claim [1], or whether it only marked the entry of the United States into a war declared in 1946 [2] or in 1923 [3] would seem to matter little today. What should matter today is the state of the war on cancer after 45, 70 or 93 years of struggle. At first glance, the struggle is not going well. Far from being conquered, cancer is now or soon will be the leading cause of death in United States and the probability an individual will die from cancer is increasing and not decreasing (Table 1) [4,5,6,7].

If the increasing annual number of deaths and the crude death rate depicted in Table 1 suggest the war on cancer is far from won, they also hide dramatic advances in understanding and avoiding certain risks, changes in demographics, and progress in diagnosis and treatment of cancer and other conditions. Because the incidence of cancer increases strikingly with age, age-adjusted death rates rather than crude death rates are now usually used and this rate for many cancers has not increased. Of greater importance, however, has been the increasing awareness of cancer and increasingly incisive approaches to diagnosis of cancer. Thus, many cancers detected today would have escaped detection a few decades ago and some cancers, such as those associated with smoking, exposure to sun and transmission of viruses, are prevented. Still, one premise for waging a war on cancer was the hope that a “magic bullet” might be found that would cure or even prevent most cancers [8,9] and yet this hope seems to have suffered more than it has profited from recent advances [10]. Here we consider the questions of whether some or many cancers might be triggered by cell fusion and whether the process of cell fusion could offer a novel target for prevention of common cancers. Put in another way, we ask whether effective targeting of cell fusion could offer a significant victory in the war on cancer.

While new therapeutics often prolong survival and even cure some cancers, Table 1 suggests eradication of cancer cannot be achieved by therapeutics because different tumors demand different therapies and because a patient cured of one cancer becomes susceptible to others. Some therapeutics, particularly immunotherapies, yield encouraging results for treating certain patients with otherwise intractable malignancies [9,11,12], however these therapies are not a “magic bullet.” Immunotherapy involves generating (or engineering) immunity to tumor antigens that differ between tumors and sometimes between individuals with the same tumor. Even if a common antigen were found, the presentation would vary with Human Leukocyte Antigen (HLA) alleles.

One might imagine that the best way to eradicate cancer would be to attack it at its inception. However, recent advances might make this approach seem increasingly remote. Some now believe many and perhaps most cancers arise when random spontaneous mutations disrupt in series the key cellular functions that distinguish normal and transformed cells [13,14,15,16,17,18]. While environmental factors, such as smoking, infection with oncogenic viruses and sunlight can increase the frequency of spontaneous mutation, and hence of some cancers, an underlying risk aligning with baseline cell proliferation remains, increasing with age. An idea has emerged that the preponderance of cancers in general or at least the relative susceptibility to cancer in various tissues predominantly reflects the number of stem cell divisions, necessarily engendering random errors of DNA copying, needed for normal development and function of a tissue [19,20]. If the analysis leading to that conclusion has sparked dispute on some points [21,22,23,24,25], it has also underscored the grim possibility that these cancers arise by happenstance (bad luck) during physiologic processes and that preventing development of these cancers can be accomplished only by disrupting normal processes (i.e., processes in many normal individuals who would not develop cancer would have to be disrupted to avert cancer in a few individuals) [26].

We shall consider whether and how often cell fusion might explain the genesis of cancer and in doing so link events external to cells with DNA damage or with random errors in replication and/or repair and whether manipulation of the propensity of cells to fuse might offer an attractive target for strategies aimed at preventing cancer or treating cancer at the outset. We think published work suggests that cell fusion *can* cause genetic changes and cancer [27,28]. Whether cell fusion actually *does* cause cancer, how often cell fusion does so and by what mechanisms remain to be determined. We shall consider these questions. If cell fusion does indeed cause cancer, it would be reasonable to question whether a therapeutic agent or a strategy that could halt the fusion of cells might appreciably lower the burden of cancer in society. We shall discuss that question as well.

## 2. Cell Fusion in Health and Cancer

Developmental and environmental factors sometimes cause cells to fuse [29,30,31,32,33]. Tight cellular and molecular regulation prevents inopportune fusion and deletes untoward progeny [32,33,34]. If one or both fusion partners previously underwent malignant transformation, the hybrid can exhibit heritable genetic and cytogenetic changes and changes in population dynamics and behavior that characterize cancer and cancer progression [35,36,37,38,39,40,41,42,43]. Some cancers can indeed be shown to contain hybrid cells [44,45,46] and some evidence suggests cancer cells might have a greater propensity than normal cells to fuse [47,48,49]. We shall be eager to learn from those who study the impact of cell fusion on cancer progression how often the capacity of cells to fuse actually arises in existing cancers; however, we shall not consider such questions here. Instead, we shall focus on whether and how the fusion of normal cells might initiate cancer and conversely whether cell fusion at the inception of cancer might also promote resistance to oncogenesis. Because cell fusion generates tetraploidy, it potentially might cause chromosomal instability, genomic plasticity and trans-differentiation thought to underlie the inception of cancer [27,28,38]. However, cell fusion has never been proved to cause malignant transformation of normal cells, except after the cells were partly transformed by oncogenic viruses [27] or in our own work, which we describe below. Thus, the key question, from our perspective is whether cell fusion or other definable and preventable cellular processes, such as aberrant mitosis, explain the preponderance of cancers that afflict members of modern societies.

## 3. Our Interest in Cell Fusion

Our interest in cell fusion and cancer began about 12 years ago when we explored what we then considered, correctly or incorrectly, to be the foremost challenge in clinical immunology—finding a way to rebuild an adaptive immune system after it had been decimated by acquired immunodeficiency disease, cancer chemotherapy or efforts to induce immune tolerance.

Rebuilding an adaptive immune system should, in principle, depend on restoring the dimensions and diversity of the B lymphocyte and T lymphocyte compartments. However, since some protective functions of B lymphocytes can be replaced by administration of gamma globulin, we assumed the limiting process in immune reconstitution was the reconstitution of the T lymphocyte repertoire. Since T cells best recognize antigen presented by the individual’s Major histocompatibility complex (MHC) encoded proteins, the T cell receptor repertoire must recognize the MHC of the individual to be restored. Since T lymphocytes develop and undergo selection in the thymus, which atrophies with age, we considered that availability of thymus and not availability of precursors for T cells limit reconstitution. Therefore, to test whether we could generate human thymocytes and potentially human T cells, we introduced human hematopoietic stem cells into fetal pigs [50], which, having an immature immune system, might harbor these cells rather than destroying them [51,52,53].

The experiments were a success. The porcine thymus was found to contain human thymocytes and the peripheral blood contained a diverse repertoire (but scarce number) of human T cells [50]. Importantly, the human T cells responded to antigen presented by antigen presenting cells from the stem cell source. What we did not expect, however, was that besides originating and selecting new T cells, the peripheral blood of the pigs contained some mononuclear cells that expressed both porcine and human proteins, contained porcine and human genes, and had chromosomes with both human and porcine DNA [54]. The hybrid cells were not “end stage” but had the capacity to proliferate and indeed the numbers increased, albeit slowly, over time. The hybrid cells were apparently selected (presumably by natural killer or NK cells) for expression or non-expression of HLA class I. Thus, some human and swine cells had fused and analysis of the karyotypes indicated that the chromosomes had recombined to form novel genomes. The formation of inter-species hybrids was of great interest to us because it suggested potential mechanisms for hastening viral and eukaryotic evolution and for viral transfer [29]. The interspecies hybrids also suggested a potential pathway to malignant transformation, i.e., via aneuploidy and DNA damage or via accelerated mutation or both and selection.

## 4. From Cell Fusion to Oncogenesis

Since our work showed that fusion of cells and co-integration of chromosomal DNA can occur spontaneously between species [54], we wondered whether fusion of isologous, non-transformed cells could also produce the chromosomal changes so prominent in inter-species hybrids and potentially malignant transformation [28]. To address that question, we used polyethylene glycol (PEG) deliberately to fuse differentially labeled intestinal epithelial cells (IEC-6 cells), which are taken to model normal, non-transformed intestinal epithelial cells [55,56,57,58,59,60,61].

After treatment with PEG, IEC-6 cells were sorted by fluorescence activated cell sorting (FACS) to separate cells containing a nucleus from differentially labeled parental cells. Approximately 60% of these cells underwent mitosis and approximately 20% were able to establish clones that contained one nucleus. The clones exhibited a range of karyotypes, indicating that fusion had established heterogeneous populations of cells. Approximately 40% of the clones were aneuploid, whereas clones generated from cells that had not fused were predominantly diploid. The cells in fusion-derived clones often had double strand DNA breaks. Thus 30%–50% of these cells were stained prominently with antibodies against histone γH2AX and/or formed tails in in single-cell gel electrophoresis or COMET assays. The cells with DNA damage were not apoptotic however as few contained activated caspases. Thus, cell fusion had generated a heterogeneous population of aneuploid cells that were proliferating and had prominent evidence of DNA damage and potentially resistance to apoptosis. As the clones expanded, the karyotypes of some reverted toward diploidy while others remained stably aneuploid. However, further diversification was not apparent, suggesting that the process that had induced aneuploidy occurred within a few cell divisions after the fusion event.

**Figure 1:**
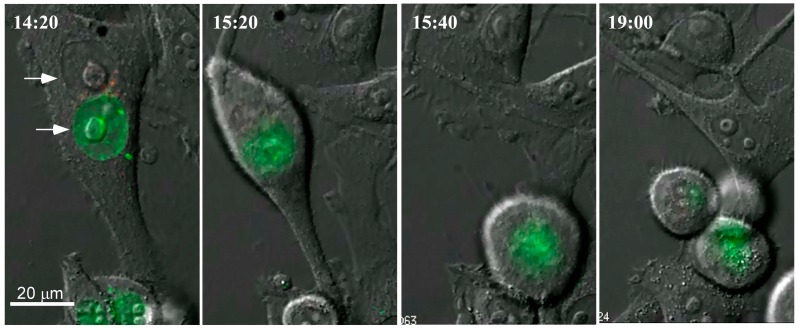
Mechanism of formation of hybrid mononuclear cells after cell fusion. HeLa cells were differentially labeled with Cyanine 5 (Cy5)-dUTP) or cyanine 3 (Cy3)-dUTP and then fused with polyethylene glycol. The first image obtained 14 h and 40 min after exposure to PEG shows a cell that has undergone fusion, the arrows denoting nuclei from parental cells. The cell contains distinct nuclei from two parental cells. At 15 h 20 min, the cell is undergoing mitosis. At 15 h 40 min, the nuclear envelopes of the parental cells have disintegrated and the labeled DNA of parental cells is admixed. At 19:00, mitosis is complete, cytokinesis is nearly complete and labeled DNA from both cells originally fused is seen in both daughter cells. This figure shows that intermixing of parental DNA occurs during but not before mitosis.

A question we considered of importance in understanding the fate of fused cells was how the cells formed by fusion of distinct parental cells resolved the condition of tetraploidy. We considered various possibilities, including the shedding of one nucleus and the fusion of the two nuclei [29]. To address this question, we biosynthetically labeled DNA in HeLa cells and fused mixtures of differentially cells with PEG, following the fate of labeled DNA by microscopy [28]. The experiment, illustrated in Figure 1, revealed clearly that as long as the nuclei remained intact, parental DNA remained separate. However, when the tetraploid cells underwent mitosis and the nuclear envelope dissolved, parental DNA intermixed. Thus, the intermixing and recombination of the tetraploid genome depended absolutely on mitosis and mitosis provided the essential prelude to aneuploidy and likely to DNA damage. We shall return to this point later when we discuss whether and how cell fusion could trigger development of cancer in vivo.

Besides exhibiting aneuploidy, DNA damage and resistance to apoptosis so often observed in malignant cells, cells that had undergone fusion were often manifestly transformed. Approximately one-third of the fusion-derived clones continued to grow at confluence in cell cultures (i.e., they had lost contact inhibition) and generated colonies in soft agar (i.e., they had lost anchorage dependence).

The fusion-derived clones also had the capacity to form tumors after transfer into immunodeficient mice. Parental cells and clones prepared from non-fused cells never formed tumors. Not unexpectedly, among the cells comprising fusion-derived clones that exhibited the capacity to form colonies in soft agar (i.e., manifestly transformed cells), many also exhibited a capacity to form tumors; whereas, the fusion-derived cells that did not form colonies in soft agar never formed tumors. These findings provided compelling evidence that cell fusion, by itself, can induce malignant transformation of archetypically normal cells.

## 5. Cell Fusion in Cancer Evolution and Progression

Our investigation of cell fusion as a potential spark for oncogenesis also provided potential insight into tumor progression. Tumor progression refers to apparent changes in the behavior of a tumor. Typical changes include faster and more aggressive growth, metastasis and drug resistance. Tumor progression is often accompanied by cytogenetic and/or genetic changes and by changes in cellular morphology. Some of the most prevalent cancers are not clinically apparent until progression has occurred and it is often (but not always) cancer progression and not cancer per se that eventuates in mortality. Hence, much effort is devoted to understanding the biological basis of cancer progression and devising ways to circumvent it. Put in another way, if the war on cancer cannot be won, that is if cancer is an inevitable outcome of aging, progress in slowing or preventing progression would profoundly limit the casualties. Although we did not study and shall not discuss the potential involvement of cell fusion in progression of cancers of long standing, we did have an opportunity to explore dynamic changes in tumor growth and karyotype during the period of months after cell fusion had originated malignant transformation.

Various theories have been put forward to explain the changes in behavior, morphology and genetics accompanying or causing progression of cancer. If loss of tumor suppression or defective DNA repair accompanying the onset of cancer persists then the stepwise genetic changes some envision to originate cancer could fuel progression [62]. The loss of tumor suppression and censoring of genetic and cytogenetic aberration would also permit chromosomal instability and genomic crises [63,64] to generate diverse populations of cells from which cells exhibiting progressive changes would be selected by the microenvironment or other factors [43,65,66]. If, in fact, cancer cells have a heightened capacity to undergo fusion, then the model we used and certain of our findings offer a glimpse at tumor progression. Thus, we found that the diverse set of transformed cells generated by a fusion event underwent changes in karyotype and behavior during growth in culture and in tumors consistent with selection (i.e., the karyotypes and morphologies became less diverse). Since the cells had been cloned at the time of fusion, the diversity could only arise at the time of or after fusion. Since diversification did not continue, the genetic changes underlying transformation were relatively stable. Unfortunately, we did not explore the transformed cells and tumors beyond the point where tumor formation was ascertained. Had we studied this model longer, we might have been able to ascertain whether changes in tumor behavior correlated with de novo genomic or karyotypic plasticity and possibly whether secondary fusion events occurred.

## 6. Reflections on the Role of Cell Fusion in Oncogenesis and Cancer Progression

If cell fusion potentially initiates malignant transformation of cells and cancer progression, there remains the question mentioned at the outset whether cell fusion actually does so. Much current understanding is drawn from experiments using mutant mice or immortalized cell lines or from investigation of established cancers in all of which models tumors must achieve a certain mass to be detected and hence to be sampled. In no model can one glimpse the moment a normal cell becomes a transformed cell or sample cells at the inception of cancer [67,68,69,70]. For this reason, there has been great enthusiasm (although much controversy as well) for validating the fundamental concepts of oncogenesis derived from experimental models and from analysis of tumor samples with cancer epidemiology [71,72,73,74,75,76].

Investigation of cells in culture, exploration of the phenotypes of mice with various mutations and the analysis of DNA sequences in samples of tumors converge to support the idea that tumors arise from the accumulation of random spontaneous mutations that disrupt a finite number of “transforming genes” (i.e., driver mutations) among the vastly more numerous mutations in neutral genes (i.e., passenger mutations) [77,78,79]. This concept appears to draw further support from the relationship between age and the death rate of cancer with age, which suggests stepwise accruing of mutations as the driving force in oncogenesis [13,14]. The correlation between the tissue specific incidence of cancer and the number of stem cell divisions in various tissues lends further support to this idea [19]. However, the seminal involvement of point mutations in initiating malignant transformation remains as one of several hypotheses concerning the genesis of cancer because the limitations mentioned above preclude direct observation of the transforming event(s) and therefore how oncogenic genetic change arises, how many changes are needed and whether the changes necessarily occur in stem cells remain matters of controversy [68,80,81,82].

One alternative, if less often voiced, explanation, for the origin of genetic changes that originate cancer is that the changes arise from mishaps in resolving such chromosomal abnormalities as aneuploidy or tetraploidy. Analysis of structure and sequence of chromosomal DNA in tumors has suggested the possibility that one or more steps in the development of some cancers might involve formation of tetraploid or aneuploid cells [83,84,85,86,87,88,89,90]. Tetraploid or aneuploid cells are much more likely than normal cells to suffer chromosomal damage and genetic changes and as a result to undergo malignant transformation. Because cancer clearly allows and may promote aneuploidy and chromosomal damage, it has been impossible to determine whether and how often aneuploidy drives malignant transformation and how often it merely results from malignancy. The question most pertinent to oncogenesis however is what process(s) generate tetraploidy or aneuploidy in the first place.

Tetraploidy and aneuploidy arise either by mitosis (without cytokinesis) or by cell fusion [28,33,63]. Mitosis in liver muscle and hematopoietic cells sometimes proceeds independently of cytokinesis (i.e., endoreplication) leading to formation of multinucleated cells and the same or a similar process might occur at the inception of cancer. Certain mononuclear cells, including trophoblast cells, myoblasts, macrophages, cardiac muscle cells and hepatocytes, fuse in development or in physiology. Regardless of how tetraploidy arises, chromosomal damage can ensue, caused by subsequent abnormalities in cytokinesis or subsequent mitotic events with failure of censoring chromosomal and mitotic errors.

If we cannot know whether a given cancer was initiated by random errors in DNA synthesis or in DNA repair or by aberrant mitosis or by cell fusion, can we hazard some inferences from cancer epidemiology? To the extent that the total number of stem cell divisions in a given tissue predicts the frequency of cancer in that tissue, we can conclude, as some now do, that the epidemiology of cancer is consistent with the hypothesis that accumulation of random errors during DNA synthesis underlies cancer. However, various objections have been raised to that idea [20,21,23,24,25] and we think that the inexact alignment between the postulated frequency of stem cell divisions and the frequency of certain cancers undermines that hypothesis. Table 2 depicts the current incidence of various cancers in the United States. Cancers of the prostate, breast and lung account for approximately 40% of new cancers. It is difficult to imagine that stem cells in prostate and breast tissues underwent more than 20-fold more divisions and that stem cells in thyroid underwent more than seven-fold as many divisions as stem cells in small intestine. Consistent with this concern is work in mice revealing that cells of the small intestine harbor more mutations than cells of the large intestine, stomach or prostate [91]. Also consistent with this concern is that cell of the heart, tumors of which are rare (Table 2), have about the same frequency of mutations as cells of the small intestine [92,93].

On the other hand, investigation of DNA synthesis and repair and sensitivity to irradiation reveal profound differences between small intestine, in which cancer is rare, and large intestine and lung, in which it is common [94,95,96]. Thus, the manner by which stem cells handle mutations and not the frequency of mutations per se might determine whether genetic changes are passed to cellular progeny and hence whether oncogenesis might ensue. Cell fusion could be pertinent in this scenario, if a cell harboring a mutation were to fuse with a cell with defective repair or defective capacity to censor defects.

The epidemiology of cancer allows us to weigh the significance of random mutation and cell fusion as inciting events in cancer and ultimately suggests an alternative hypothesis for the origin of the most common malignancies. Table 2 shows that cancers of the highest incidence arise in tissues such as prostate, breast, and lung, thyroid and pancreas that have: (i) a low baseline rate of mitosis and number of cumulative cell divisions; and (ii) scarce evidence of cell fusion, as a normal process. Thus, cancer of the lung, breast, prostate, kidney, thyroid, and pancreas, in which baseline rate and number of cumulative cell divisions at maturity are low and cell fusion is not known to occur with any frequency account for approximately 56% of all malignancies. In contrast, cancers of hematopoietic cells and the gastrointestinal track, in which cell division is continuous and cell fusion does occur, account for only 24% of malignancies. Frequency of cell fusion in physiology also does not itself predict malignancy. Trophoblast, heart, and muscle, liver and myeloid cells often undergo fusion; yet, cancer originating from these cells is uncommon or rare. The liver provides an especially interesting example both of aberrant cytokinesis and cell fusion. Hepatocytes commonly undergo mitosis without cytokinesis and exhibit polyploidy and aneuploidy [97]. Hepatocytes also commonly undergo fusion with hepatocytes (homotypic) or other cells (heterotypic), generating tetraploid and polyploid cells that can undergo reduction division, potentially generating aneuploidy [98]. Yet, despite striking chromosomal instability, hepatocytes do not often undergo malignant transformation in the absence of another factor such as hepatitis virus infection or cirrhosis. Infection with oncogenic viruses commonly induces fusion of infected cells with each other or with uninfected cells [27]. However, only about 4% of new cases of cancer are caused by oncogenic viruses (Table 3) and the fraction of these cancers actually sparked by cell fusion is not known.

Most cancers thus arise in cells and tissues in which neither mitosis nor cell fusion is common. That suggests extrinsic factors rather than a random process originates the most common types of cancer. Cells that rarely proliferate and that do not commonly undergo fusion have no baseline requirement for apparatus that censors for errors in DNA synthesis or chromosomal aberration. We suspect it is precisely such cells that lack high levels of baseline tumor suppression that would be most susceptible to oncogenic events, such as untoward fusion or mitosis.

## 7. Cell Fusion in the War on Cancer

We began this essay with the discouraging proposition that advances in medicine and biomedical science have made the “war on cancer” seem impossible to win (e.g., increasing longevity increases the incidence of cancer and ascendency of random errors in DNA synthesis as a cause of cancer makes prevention of all cancers impossible). If errors in mitosis or cytokinesis that eventuate in tetraploidy and aneuploidy also occur randomly then this mechanism also undermines at least some potential strategies for prevention. On the other hand, if cell fusion initiates cancer, one might imagine at least two targets for therapeutics—the macromolecules that make fusion more energetically feasible and the factors or processes that protect cells, such as hepatocytes, from oncogenesis after fusion occurs in the normal setting. The development of such specific therapeutics would certain make it possible to test the significance of cell fusion as a mechanism of oncogenesis. But, should we hope the therapeutics could also solve the problem of cancer? We are far from certain about the answer to this question because while cell fusion might indeed explain some of the most common cancers, we can envision mechanisms though which cell fusion might protect against cancer.

Among the first observations made about cell fusion in cancer was that hybrids formed by fusion of malignant cells with non-malignant cells sometimes adopt the properties of the non-malignant partner [106,107]. Conceivably, then, malignancy might be suppressed or even reversed cancer cells fuse with normal cells. Since the properties of the non-malignant cell are postulated to include fashioning of a microenvironment inimical to malignancy, one might imagine further that presence or expansion (owing to fusion) of non-malignant cells might thwart cancer at its inception. While appealing as an explanation for the importance of the microenvironment [43,108], we find the concept unpersuasive as a mechanism of defense against cancer after an oncogenic founder has divided. Fusion of a malignant cell with a non-malignant partner might also fuel progression, as discussed above, or even transmit oncogenicity [109,110].

On the other hand, we can envision mechanisms by which cell fusion might confer protection beyond the fate of the fused cells. Cells recently fused are especially vulnerable to lysis (necrosis), perhaps owing to entry of excess Ca++ or repositioning of cytoplasmic organelles. Necrosis of tumor cells might be relatively infrequent and have little direct impact on tumor mass; however, necrosis of one or a few cells can have local or regional impact on the viability of surrounding cells [111]. Necrosis activates complement and coagulation generating hemostasis and thrombosis and promoting encapsulation, potentially circumscribing “threats.” We have referred to this form of protection as regional immunity [112]. Regional immunity is unlikely to have a profound impact on an established or advanced tumor but it could circumscribe and destroy a small cluster of recently damaged or transformed cells.

**Figure 2:**
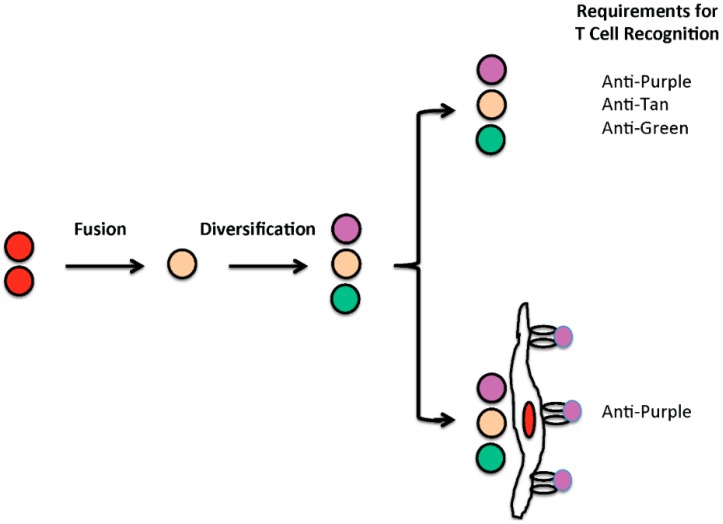
Impact of cell fusion on tumor immune surveillance mediated by T cells. Cell fusion potentially causes genomic diversification, modeled by conversion of red cells with red protein to cells with purple, tan or green proteins. An individual is tolerant to the native, red protein, but the immune system, i.e., the T cell repertoire, potentially recognizes purple, tan or green variants. In principle, diversification can spark immunity to some variants. However, extensive diversification, as modeled at the top, might allow tumor cells to escape immune control unless all variants are recognized. On the other hand, where tumor protein variants are taken up and presented by endothelial cells, as modeled below, recognition of one or a few highly expressed variants might suffice to support T cell mediated control of tumor growth. In this later case T cell interaction with capillaries activates or destroys endothelium causing tumor cells to be circumscribed or killed.

Cell fusion also potentially facilitates immune surveillance against tumors. Changes in coding sequences, whether driver or passenger, potentially generate novel “tumor” antigens and changes in regulatory sequences potentially increase the production of autoantigens. Of course, many tumor cells will not produce the neoantigen or an autoantigen; but, abundant neoantigen or autoantigen released from a necrotic tumor cell might be taken up and presented by antigen presenting cells. Those who study the immunology of transplants have known for decades, that blood vessels in the vicinity of “foreign” cells effectively take up and present foreign antigens and that it is immunity targeting those blood vessels that destroys cellular (tumor) grafts (Figure 2) [113,114]. Thus, protein variants produced by one or a few cells can generate and serve as a target of powerful immunity directed against the capillaries feeding those cells and the activation or destruction of small blood vessel endothelium circumscribes and/or starves tumor cells in the vicinity of the affected blood vessels. In this way cell fusion might actually spark anti-tumor immunity that destroys a small initial focus of cancer cells.

Thus, while the development of therapeutics that disrupt the untoward fusion of cells could shed light on the relative contribution of cell fusion to the origination of tumors, the therapeutics could also prove to be a pyrrhic victory in the war on cancer if cell fusion is vital to resistance against tumor growth. However, we would then know the function of cell fusion in malignancy. Regardless, we look forward to the development of such tools.

## Figures and Tables

**Table 1:** Cancer deaths and death rates in U.S. 1923–2013 *.

Year	US Population × 10^6^	Number of Cancer Deaths	Crude Death Rate
2013	315	611,105	193
1971	208	330,730	163
1946	141	182,005	130
1923	112	85,575	88

* Adapted from Xu et al. National vital statistics report, Volume 64, Number 2, 2016 [7].

**Table 2:** Incidence of selected cancers in the United States in 2013 *.

Cancer	New Cases (2013)	Percent of Total
Prostate	238,590	14.37
Breast	234,580	14.13
Lung	228,190	13.74
Lymphoid **	123,130	7.42
Colon	102,480	6.17
Kidney	65,150	3.92
Thyroid	60,220	3.63
Pancreas	45,220	2.72
Liver	30,640	1.85
Myeloid	20,510	1.24
Muscle ^#^	11,410	0.07
Small intestine	8810	0.05
Cardiac ^+^	102	0.00006
Total new cancers ^	1,660,290	

* Adapted from Siegel et al. [6]. The number of new cases excludes basal cell and squamous cell carcinoma of skin and in situ carcinomas; ** Includes lymphoma, multiple myeloma, acute lymphocytic leukemia and chronic lymphocytic leukemia; ^#^ Muscle and other soft tissues, including cardiac which are listed separately below; ^+^ Based on the NIH Surveillance, Epidemiology and End Results Program (SEER) frequency of soft tissue sarcoma between 1988 and 2005, expressed per year; ^ Total includes 491,360 cancers of types not listed in the table.

**Table 3:** Frequency of cancers associated with human oncogenic viruses that can induce cell fusion [27,99] ^+^.

Virus	Affected Cells	Cancer	Frequency (Estimated)	Reference
Human papillomavirus	epithelial cells	cervical	12,900	[100]
		anal	7270	
		oropharyngeal	15,520	
Hepatitis C, hepatitis B	hepatocytes	hepatoma	30,640 ^#^	[101]
HIV *	T cells			[102]
	endothelial cells	Kaposi (soft tissue) sarcoma	1943	
HHV-8	endothelial cells	Kaposi sarcoma	1943	[103]
HTLV-1	T cells	T cell leukemia	rare	[104]
EBV	B cells	Burkitt’s	1652	[105]
	epithelial cells	Lymphoma Nasopharyngeal	3200	
Total virus associated			73,125	
Total new cancers *			1,660,290	

^+^ Estimated frequency of new cases in the U.S. extrapolated from Siegel et al. [6] and other sources. Since HIV and HHV-8 are associated with Kaposi sarcoma, the total of new cases is listed for both viruses. For reviews see [27,99]; * HIV, human immunodeficiency virus-1; HHV, human herpesvirus; EBV, Epstein-Barr virus; ^#^ Total cases per year; most but not all are linked to infection with hepatitis viruses.

## References

[1] Price D.E. (1978). The politics of the war on cancer. Science.

[2] Stebbing G.F. (1946). Total war on cancer. Br. Med. J..

[3] Dukes C.E. (1965). The origin and early history of the imperial cancer research fund. Ann. R. Coll. Surg. Engl..

[4] Epstein S.S. (1990). Losing the war against cancer: Who’s to blame and what to do about it. Int. J. Health Serv..

[5] Hoyert D.L. (2012). 75 years of mortality in the United States, 1935–2010. NCHS Data Brief, No. 88.

[6] Siegel R.L., Miller K.D., Jemal A. (2015). Cancer statistics, 2015. CA Cancer J. Clin..

[7] Xu J., Murphy S.L., Kochanek K.D., Bastian B.A. (2016). Deaths: Final data for 2013. Natl. Vital Stat. Rep..

[8] Cornell D.H. (1997). The war on cancer: From the benign to the malignant. N. Engl. J. Med..

[9] Topalian S.L., Wolchok J.D., Chan T.A., Mellman I., Palucka K., Banchereau J., Rosenberg S.A., Dane Wittrup K. (2015). Immunotherapy: The path to win the war on cancer?. Cell.

[10] Hanahan D. (2014). Rethinking the war on cancer. Lancet.

[11] Littman D.R. (2015). Releasing the brakes on cancer immunotherapy. Cell.

[12] Koch M. (2016). Cancer immunotherapy booster. Cell.

[13] Nordling C.O. (1953). A new theory on cancer-inducing mechanism. Br. J. Cancer.

[14] Armitage P., Doll R. (1954). The age distribution of cancer and a multi-stage theory of carcinogenesis. Br. J. Cancer.

[15] Land H., Parada L.F., Weinberg R.A. (1983). Tumorigenic conversion of primary embryo fibroblasts requires at least two cooperating oncogenes. Nature.

[16] Hanahan D., Weinberg R.A. (2011). Hallmarks of cancer: The next generation. Cell.

[17] Vogelstein B., Kinzler K.W. (2015). The path to cancer—Three strikes and you’re out. N. Engl. J. Med..

[18] Tomasetti C., Marchionni L., Nowak M.A., Parmigiani G., Vogelstein B. (2015). Only three driver gene mutations are required for the development of lung and colorectal cancers. Proc. Natl. Acad. Sci. USA.

[19] Tomasetti C., Vogelstein B. (2015). Cancer etiology. Variation in cancer risk among tissues can be explained by the number of stem cell divisions. Science.

[20] Crossan G.P., Garaycoechea J.I., Patel K.J. (2015). Do mutational dynamics in stem cells explain the origin of common cancers?. Cell Stem Cell.

[21] Rozhok A.I., Wahl G.M., deGregori J. (2015). A critical examination of the “bad luck” explanation of cancer risk. Cancer Prev. Res..

[22] Belpomme D., Irigaray P. (2016). Replicative random mutations as an unproven cause of cancer: A technical comment. Mol. Clin. Oncol..

[23] Giovannucci E.L. (2016). Are most cancers caused by specific risk factors acting on tissues with high underlying stem cell divisions?. J. Natl. Cancer Inst..

[24] Little M.P., Hendry J.H., Puskin J.S. (2016). Lack of correlation between stem-cell proliferation and radiation- or smoking-associated cancer risk. PLoS ONE.

[25] Wu S., Powers S., Zhu W., Hannun Y.A. (2016). Substantial contribution of extrinsic risk factors to cancer development. Nature.

[26] Albini A., Cavuto S., Apolone G., Noonan D.M. (2015). Strategies to prevent “bad luck” in cancer. J. Natl. Cancer Inst..

[27] Duelli D.M., Padilla-Nash H.M., Berman D., Murphy K.M., Ried T., Lazebnik Y. (2007). A virus causes cancer by inducing massive chromosomal instability through cell fusion. Curr. Biol..

[28] Zhou X., Merchak K., Lee W., Grande J.P., Cascalho M., Platt J.L. (2015). Cell fusion connects oncogenesis with tumor evolution. Am. J. Pathol..

[29] Ogle B.M., Cascalho M., Platt J.L. (2005). Biological implications of cell fusion. Nat. Rev. Mol. Cell Biol..

[30] Baker J.R. (1953). The cell theory: A restatement, history and critique. Part IV. The multiplication of cells. Q. J. Microsc. Sci..

[31] Wolpert L. (1996). The evolution of ‘the cell theory’. Curr. Biol. CB.

[32] Oren-Suissa M., Podbilewicz B. (2007). Cell fusion during development. Trends Cell Biol..

[33] Zhou X., Platt J.L. (2011). Molecular and cellular mechanisms of mammalian cell fusion. Adv. Exp. Med. Biol..

[34] Helming L., Gordon S. (2009). Molecular mediators of macrophage fusion. Trends Cell Biol..

[35] Barski G., Sorieul S., Cornefert F. (1961). “Hybrid” type cells in combined cultures of two different mammalian cell strains. J. Natl. Cancer Inst..

[36] Sorieul S., Ephrussi B. (1961). Karyological demonstration of hybridization of mammalian cells in vitro. Nature.

[37] Harris H. (1971). Cell fusion and the analysis of malignancy. Proc. R. Soc. Lond. Ser. B Biol. Soc..

[38] Bjerkvig R., Tysnes B.B., Aboody K.S., Najbauer J., Terzis A.J. (2005). Opinion: The origin of the cancer stem cell: Current controversies and new insights. Nat. Rev. Cancer.

[39] Jacobsen B.M., Harrell J.C., Jedlicka P., Borges V.F., Varella-Garcia M., Horwitz K.B. (2006). Spontaneous fusion with, and transformation of mouse stroma by, malignant human breast cancer epithelium. Cancer Res..

[40] Lu X., Kang Y. (2009). Cell fusion as a hidden force in tumor progression. Cancer Res..

[41] Powell A.E., Anderson E.C., Davies P.S., Silk A.D., Pelz C., Impey S., Wong M.H. (2011). Fusion between intestinal epithelial cells and macrophages in a cancer context results in nuclear reprogramming. Cancer Res..

[42] Berndt B., Zanker K.S., Dittmar T. (2013). Cell fusion is a potent inducer of aneuploidy and drug resistance in tumor cell/normal cell hybrids. Crit. Rev. Oncog..

[43] Dittmar T., Zanker K.S. (2015). Tissue regeneration in the chronically inflamed tumor environment: Implications for cell fusion driven tumor progression and therapy resistant tumor hybrid cells. Int. J. Mol. Sci..

[44] Chakraborty A., Lazova R., Davies S., Backvall H., Ponten F., Brash D., Pawelek J. (2004). Donor DNA in a renal cell carcinoma metastasis from a bone marrow transplant recipient. Bone Marrow Transplant..

[45] Yilmaz Y., Lazova R., Qumsiyeh M., Cooper D., Pawelek J. (2005). Donor y chromosome in renal carcinoma cells of a female bmt recipient: Visualization of putative bmt-tumor hybrids by fish. Bone Marrow Transplant..

[46] Lazova R., Laberge G.S., Duvall E., Spoelstra N., Klump V., Sznol M., Cooper D., Spritz R.A., Chang J.T., Pawelek J.M. (2013). A melanoma brain metastasis with a donor-patient hybrid genome following bone marrow transplantation: First evidence for fusion in human cancer. PLoS ONE.

[47] Goldenberg D.M., Gotz H. (1968). On the ‘human’ nature of highly malignant heterotransplantable tumors of human origin. Eur. J. Cancer.

[48] Miller F.R., McInerney D., Rogers C., Miller B.E. (1988). Spontaneous fusion between metastatic mammary tumor subpopulations. J. Cell. Biochem..

[49] Rachkovsky M., Sodi S., Chakraborty A., Avissar Y., Bolognia J., McNiff J.M., Platt J., Bermudes D., Pawelek J. (1998). Melanoma × macrophage hybrids with enhanced metastatic potential. Clin. Exp. Metastasis.

[50] Ogle B.M., Knudsen B.E., Nishitai R., Ogata K., Platt J.L. (2009). Toward the development of human T cells in swine for potential use in adoptive T cell immunotherapy. Tissue Eng..

[51] Zanjani E.D., Pallavicini M.G., Ascensao J.L., Flake A.W., Langlois R.G., Reitsma M., MacKintosh F.R., Stutes D., Harrison M.R., Tavassoli M. (1992). Engraftment and long-term expression of human fetal hemopoietic stem cells in sheep following transplantation in utero. J. Clin. Investig..

[52] Zanjani E.D., Flake A.W., Rice H., Hedrick M., Tavassoli M. (1994). Long-term repopulating ability of xenogeneic transplanted human fetal liver hematopoietic stem cells in sheep. J. Clin. Investig..

[53] Liechty K.W., MacKenzie T.C., Shaaban A.F., Radu A., Moseley A.M., Deans R., Marshak D.R., Flake A.W. (2000). Human mesenchymal stem cells engraft and demonstrate site-specific differentiation after in utero transplantation in sheep. Nat. Med..

[54] Ogle B.M., Butters K.A., Plummer T.B., Ring K.R., Knudsen B.E., Litzow M.R., Cascalho M., Platt J.L. (2004). Spontaneous fusion of cells between species yields transdifferentiation and retroviral transfer in vivo. FASEB J..

[55] Quaroni A., Wands J., Trelstad R.L., Isselbacher K.J. (1979). Epithelioid cell cultures from rat small intestine. Characterization by morphologic and immunologic criteria. J. Cell Biol..

[56] Thomas C., Oates P.S. (2002). IEC-6 cells are an appropriate model of intestinal iron absorption in rats. J. Nutr..

[57] Ouko L., Ziegler T.R., Gu L.H., Eisenberg L.M., Yang V.W. (2004). Wnt11 signaling promotes proliferation, transformation, and migration of IEC6 intestinal epithelial cells. J. Biol. Chem..

[58] Boucher M.J., Jean D., Vezina A., Rivard N. (2004). Dual role of MEK/ERK signaling in senescence and transformation of intestinal epithelial cells. Am. J. Physiol. Gastrointest. Liver Physiol..

[59] Nandan M.O., McConnell B.B., Ghaleb A.M., Bialkowska A.B., Sheng H., Shao J., Babbin B.A., Robine S., Yang V.W. (2008). Kruppel-like factor 5 mediates cellular transformation during oncogenic kras-induced intestinal tumorigenesis. Gastroenterology.

[60] Voisin L., Julien C., Duhamel S., Gopalbhai K., Claveau I., Saba-El-Leil M.K., Rodrigue-Gervais I.G., Gaboury L., Lamarre D., Basik M. (2008). Activation of MEK1 or MEK2 isoform is sufficient to fully transform intestinal epithelial cells and induce the formation of metastatic tumors. BMC Cancer.

[61] Duhamel S., Hebert J., Gaboury L., Bouchard A., Simon R., Sauter G., Basik M., Meloche S. (2012). Sef downregulation by ras causes MEK1/2 to become aberrantly nuclear localized leading to polyploidy and neoplastic transformation. Cancer Res..

[62] Swanton C. (2015). Cancer evolution constrained by mutation order. N. Engl. J. Med..

[63] Davoli T., de Lange T. (2011). The causes and consequences of polyploidy in normal development and cancer. Annu. Rev. Cell Dev. Biol..

[64] Dewhurst S.M., McGranahan N., Burrell R.A., Rowan A.J., Gronroos E., Endesfelder D., Joshi T., Mouradov D., Gibbs P., Ward R.L. (2014). Tolerance of whole-genome doubling propagates chromosomal instability and accelerates cancer genome evolution. Cancer Discov..

[65] Junttila M.R., de Sauvage F.J. (2013). Influence of tumour micro-environment heterogeneity on therapeutic response. Nature.

[66] Aktipis C.A., Boddy A.M., Gatenby R.A., Brown J.S., Maley C.C. (2013). Life history trade-offs in cancer evolution. Nat. Rev. Cancer.

[67] Liu C., Sage J.C., Miller M.R., Verhaak R.G., Hippenmeyer S., Vogel H., Foreman O., Bronson R.T., Nishiyama A., Luo L. (2011). Mosaic analysis with double markers reveals tumor cell of origin in glioma. Cell.

[68] Visvader J.E. (2011). Cells of origin in cancer. Nature.

[69] Blyth K., Morton J.P., Sansom O.J. (2012). The right time, the right place: Will targeting human cancer-associated mutations to the mouse provide the perfect preclinical model?. Curr. Opin. Genet. Dev..

[70] Zong Y., Goldstein A.S., Witte O.N. (2015). Tissue recombination models for the study of epithelial cancer. Cold Spring Harb. Protoc..

[71] Cairns J. (1975). Mutation selection and the natural history of cancer. Nature.

[72] Doll R. (1978). An epidemiological perspective of the biology of cancer. Cancer Res..

[73] Armitage P. (1985). Multistage models of carcinogenesis. Environ. Health Perspect..

[74] Peto J. (2001). Cancer epidemiology in the last century and the next decade. Nature.

[75] Meza R., Jeon J., Moolgavkar S.H., Luebeck E.G. (2008). Age-specific incidence of cancer: Phases, transitions, and biological implications. Proc. Natl. Acad. Sci. USA.

[76] Noble R., Kaltz O., Hochberg M.E. (2015). Peto’s paradox and human cancers. Philos. Trans. R. Soc. Lond. B Biol. Sci..

[77] Vogelstein B., Fearon E.R., Hamilton S.R., Kern S.E., Preisinger A.C., Leppert M., Nakamura Y., White R., Smits A.M., Bos J.L. (1988). Genetic alterations during colorectal-tumor development. N. Engl. J. Med..

[78] Vogelstein B., Papadopoulos N., Velculescu V.E., Zhou S., Diaz L.A., Kinzler K.W. (2013). Cancer genome landscapes. Science.

[79] Ortmann C.A., Kent D.G., Nangalia J., Silber Y., Wedge D.C., Grinfeld J., Baxter E.J., Massie C.E., Papaemmanuil E., Menon S. (2015). Effect of mutation order on myeloproliferative neoplasms. N. Engl. J. Med..

[80] Rosen J.M., Jordan C.T. (2009). The increasing complexity of the cancer stem cell paradigm. Science.

[81] Tomasson M.H. (2009). Cancer stem cells: A guide for skeptics. J. Cell Biochem..

[82] Visvader J.E., Lindeman G.J. (2010). Stem cells and cancer—The promise and puzzles. Mol. Oncol..

[83] Fujiwara T., Bandi M., Nitta M., Ivanova E.V., Bronson R.T., Pellman D. (2005). Cytokinesis failure generating tetraploids promotes tumorigenesis in p53-null cells. Nature.

[84] Castedo M., Coquelle A., Vivet S., Vitale I., Kauffmann A., Dessen P., Pequignot M.O., Casares N., Valent A., Mouhamad S. (2006). Apoptosis regulation in tetraploid cancer cells. EMBO J..

[85] Ganem N.J., Godinho S.A., Pellman D. (2009). A mechanism linking extra centrosomes to chromosomal instability. Nature.

[86] Ganem N.J., Cornils H., Chiu S.Y., O’Rourke K.P., Arnaud J., Yimlamai D., Thery M., Camargo F.D., Pellman D. (2014). Cytokinesis failure triggers hippo tumor suppressor pathway activation. Cell.

[87] Lim S., Ganem N.J. (2014). Tetraploidy and tumor development. Oncotarget.

[88] Lissa D., Senovilla L., Rello-Varona S., Vitale I., Michaud M., Pietrocola F., Boileve A., Obrist F., Bordenave C., Garcia P. (2014). Resveratrol and aspirin eliminate tetraploid cells for anticancer chemoprevention. Proc. Natl. Acad. Sci. USA.

[89] Stephens P.J., Greenman C.D., Fu B., Yang F., Bignell G.R., Mudie L.J., Pleasance E.D., Lau K.W., Beare D., Stebbings L.A. (2011). Massive genomic rearrangement acquired in a single catastrophic event during cancer development. Cell.

[90] Zhao B., Guan K.L. (2014). Hippo pathway key to ploidy checkpoint. Cell.

[91] Behjati S., Huch M., van Boxtel R., Karthaus W., Wedge D.C., Tamuri A.U., Martincorena I., Petljak M., Alexandrov L.B., Gundem G. (2014). Genome sequencing of normal cells reveals developmental lineages and mutational processes. Nature.

[92] Vijg J., Busuttil R.A., Bahar R., Dolle M.E. (2005). Aging and genome maintenance. Ann. N. Y. Acad. Sci..

[93] Busuttil R.A., Garcia A.M., Reddick R.L., Dolle M.E., Calder R.B., Nelson J.F., Vijg J. (2007). Intra-organ variation in age-related mutation accumulation in the mouse. PLoS ONE.

[94] Cairns J. (2002). Somatic stem cells and the kinetics of mutagenesis and carcinogenesis. Proc. Natl. Acad. Sci. USA.

[95] Potten C.S., Owen G., Booth D. (2002). Intestinal stem cells protect their genome by selective segregation of template DNA strands. J. Cell Sci..

[96] Gandara R.M., Mahida Y.R., Potten C.S. (2012). Regional differences in stem and transit cell proliferation and apoptosis in the terminal ileum and colon of mice after 12 Gy. Int. J. Radiat. Oncol. Biol. Phys..

[97] Duncan A.W., Taylor M.H., Hickey R.D., Hanlon Newell A.E., Lenzi M.L., Olson S.B., Finegold M.J., Grompe M. (2010). The ploidy conveyor of mature hepatocytes as a source of genetic variation. Nature.

[98] Duncan A.W., Hickey R.D., Paulk N.K., Culberson A.J., Olson S.B., Finegold M.J., Grompe M. (2009). Ploidy reductions in murine fusion-derived hepatocytes. PLoS Genet..

[99] McLaughlin-Drubin M.E., Munger K. (2008). Viruses associated with human cancer. Biochim. Biophys. Acta.

[100] Hu L., Plafker K., Vorozhko V., Zuna R.E., Hanigan M.H., Gorbsky G.J., Plafker S.M., Angeletti P.C., Ceresa B.P. (2009). Human papillomavirus 16 E5 induces bi-nucleated cell formation by cell-cell fusion. Virology.

[101] Gao P., Zheng J. (2011). Oncogenic virus-mediated cell fusion: New insights into initiation and progression of oncogenic viruses-related cancers. Cancer Lett..

[102] Roy N.H., Chan J., Lambele M., Thali M. (2013). Clustering and mobility of HIV-1 Env at viral assembly sites predict its propensity to induce cell-cell fusion. J. Virol..

[103] Pertel P.E. (2002). Human herpesvirus 8 glycoprotein B (gB), gH, and gL can mediate cell fusion. J. Virol..

[104] Hoshino H., Shimoyama M., Miwa M., Sugimura T. (1983). Detection of lymphocytes producing a human retrovirus associated with adult T-cell leukemia by syncytia induction assay. Proc. Natl. Acad. Sci. USA.

[105] Bayliss G.J., Wolf H. (1981). An Epstein—Barr virus early protein induces cell fusion. Proc. Natl. Acad. Sci. USA.

[106] Harris H., Miller O.J., Klein G., Worst P., Tachibana T. (1969). Suppression of malignancy by cell fusion. Nature.

[107] Harris H., Bock G., Marsh J. (1989). The Biology of Tumour Suppression. Genetic Analysis of Tumor Suppression.

[108] Winton D.J., Blount M.A., Ponder B.A. (1989). Polyclonal origin of mouse skin papillomas. Br. J. Cancer.

[109] Goldenberg D.M., Rooney R.J., Loo M., Liu D., Chang C.H. (2014). In-vivo fusion of human cancer and hamster stromal cells permanently transduces and transcribes human DNA. PLoS ONE.

[110] Lazebnik Y. (2014). The shock of being united: Another lesson from plants?. Cell Cycle.

[111] Platt J.L., Cascalho M. (2015). IgM in the kidney: A multiple personality disorder. Kidney Int..

[112] Saadi S., Wrenshall L.E., Platt J.L. (2002). Regional manifestations and control of the immune system. FASEB J..

[113] Cascalho M., Platt J.L. (2006). The immunologic barriers to replacing damaged organs. Curr. Immunol. Rev..

[114] Platt J.L., Cascalho M., Mulholland M.W., Lillemoe K.D., Doherty G.M., Maier R.V., Simeone D.M., Upchurch G.R.J. (2011). Transplantation immunology. Greenfield’s Surgery: Scientific Principles and Practice.

